# Chemically Enhanced Immunogenicity of Bacteria by Supramolecular Functionalization with an Adjuvant

**DOI:** 10.1002/cbic.202200434

**Published:** 2022-11-04

**Authors:** Nikolas Duszenko, Danny M. van Willigen, Anton Bunschoten, Aldrik H. Velders, Meta Roestenberg, Fijs W. B. van Leeuwen

**Affiliations:** ^1^ Interventional Molecular Imaging (IMI) Laboratory Department of Radiology Leiden University Medical Center Albinusdreef 2 2333 RC Leiden The Netherlands; ^2^ Interventional Molecular Imaging (IMI) Laboratory Departments of Radiology & Parasitology Leiden University Medical Center Albinusdreef 2 2333 RC Leiden (The Netherlands; ^3^ Interventional Molecular Imaging (IMI) Laboratory Department of Radiology Leiden University Medical Center Albinusdreef 2 2333 RC Leiden (The Netherlands; ^4^ Laboratory of BioNanoTechnology Wageningen University & Research Bornse Weilanden 9 6708 WG Wageningen (The Netherlands; ^5^ Laboratory of BioNanoTechnology Wageningen University & Research Bornse Weilanden 9 6708 WG Wageningen (The Netherlands; ^6^ Departments of Parasitology & Infectious Diseases Leiden University Medical Center Albinusdreef 2 2333 RC Leiden (The Netherlands

**Keywords:** bacteria, conjugation, immunology, inflammation, supramolecular chemistry

## Abstract

Many pathogens blunt immune responses because they lack immunogenic structural features, which typically results in disease. Here, we show evidence suggesting that pathogen immunogenicity can be chemically enhanced. Using supramolecular host‐guest chemistry, we complexed onto the surface of a poorly immunogenic bacterium (*Staphylococcus aureus*) a TLR7 agonist‐based adjuvant. “Adjuvanted” bacteria were readily recognized by macrophages and induced a more pro‐inflammatory immunophenotype. Future applications of this concept could yield treatment modalities that bolster the immune system's response to pathogenic microbes.

## Graphical abstract

**Poorly immunogenic** pathogens are a major cause of infectious disease. We chemically modified the surface of such a pathogen (*Staphylococcus aureus*) with a synthetic adjuvant to boost immunogenicity. “Adjuvanted” *S. aureus* induced improved immune responses in macrophages compared to unadulterated bacteria.

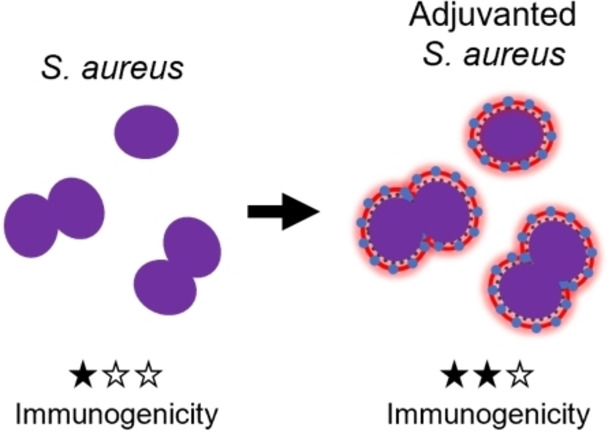

## Introduction

Immune responses to pathogens are critically dependent on recognition of pathogen‐associated molecular patterns (PAMPs).^[1]^ Sentinel immune cells such as macrophages possess a suite of receptors facilitating recognition, such as Toll‐like receptors (TLRs), activation of which triggers the development of an antimicrobial phenotype characterized by e. g. increased secretion of pro‐inflammatory cytokines.^[2]^ Accordingly, many pathogens have evolved mechanisms for avoiding such activation.^[3]^ For example, the bacterium *Staphylococcus aureus* enzymatically cleaves some of its surface components and renders them inert to immune cells, endowing it with a low immunogenicity that yields suboptimal immune activation and can lead to chronic metastatic infection and disease.^[4]^

The issue of low immunogenicity has long been known in vaccinology and led to the concept of adjuvanting: addition of exogenous immunogens called “adjuvants” to boost entities’ immunogenicity.^[5]^ Clinically registered subunit vaccines such as Prevnar and RTS,S,^[6]^ as well as next‐generation nanoparticle vaccines,^[7]^ have shown that tightly tethering immunogens tends to yield better boosts in immunogenicity. We thus reasoned that the same concept might be translated to improve (pathogenic) microbes’ immunogenicity.

Here, we show the conceptual feasibility of boosting the immunogenicity of *S. aureus* bacteria by chemically modifying the cell surface with the synthetic TLR7 agonist‐based adjuvant CL307. This adjuvant was chosen due to a number of useful properties: 1) a reactive spermine moiety for conjugating to host polymers that would facilitate recognition of the agonist by TLR7; 2) a small size (597 Da) with minimal impact on the host polymers’ chemical properties; and 3) an absorption at 298 nm that allowed for quantitation. The resulting “adjuvanted” bacteria were normally recognized by macrophages and yielded improved immune activation compared to unadulterated bacteria.

## Results and Discussion

**Figure 1:**
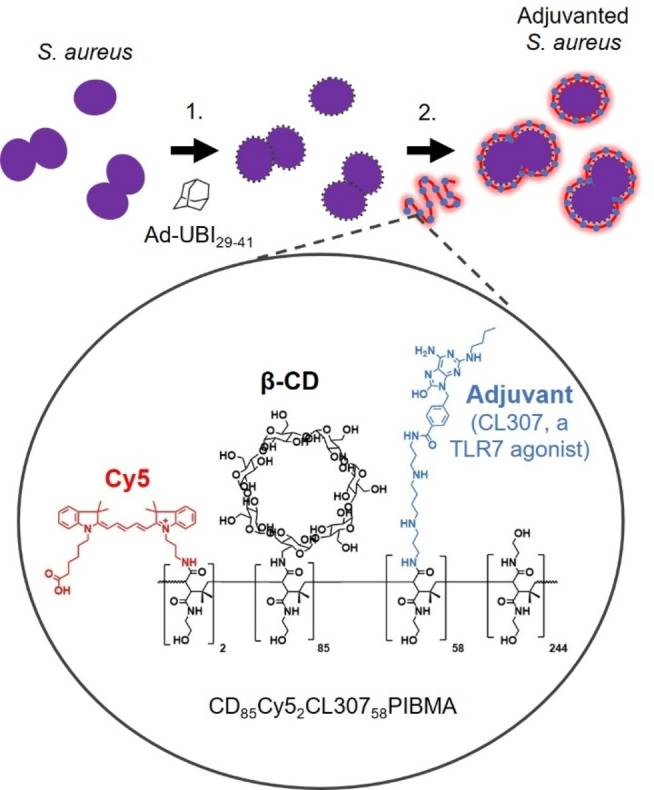
Schematic illustrating chemical strategy for introducing an adjuvant onto the surface of *S. aureus* bacteria. The bacterial surface was first pre‐functionalized with Ad using the antibacterial peptide UBI_29–41_ as a vector, followed by supramolecular complexation of CD‐ and CL307‐bearing polymers onto the cell surface. Ad=adamantane; UBI=ubiquicidin; CD=β‐cyclodextrin.

To introduce CL307 onto the surface of *S. aureus* bacteria, we adapted a supramolecular host‐guest chemistry technique for functionalizing the cell surface of live bacterial and eukaryotic cells (Figure 1).^[8]^ An initial pre‐functionalization of the surface with an adamantane (Ad) moiety was achieved via a bacteria‐targeting Ad‐UBI_29–41_ construct, as the UBI_29–41_ vector constitutes a cationic, antimicrobial peptide that inserts into the bacterial cell membrane.^[9]^ This Ad pre‐functionalization was followed by the complexation of complementary β‐cyclodextrin (CD)‐bearing PIBMA polymers that can, in addition to CD, serve as carrier for other functionalities of interest. Here, these functionalities comprised 1) Cy5 dyes for tracking the polymers, and 2) the adjuvant CL307.

**Figure 2:**
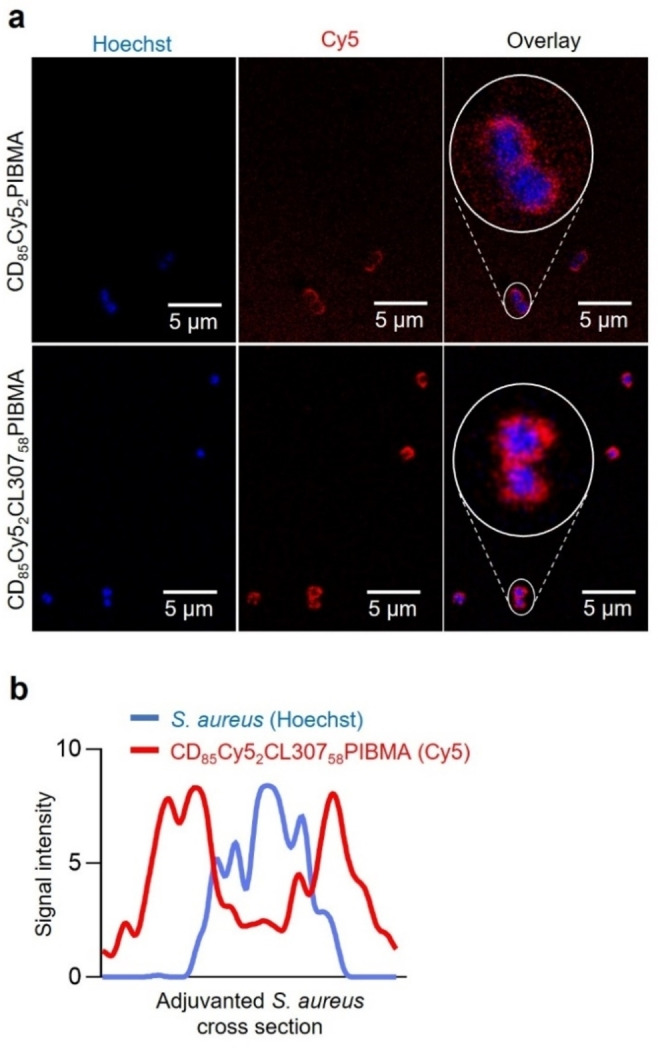
Complexation of CL307‐bearing host polymers onto the bacterial cell surface to yield adjuvanted *S. aureus*. a) Confocal fluorescence microscopy images of bacteria with complexed host polymer (top row) and CL307‐bearing host polymer (bottom row) showing Hoechst signal from the bacterial cytoplasm (left ‐ blue), Cy5 signal from complexed (CL307‐bearing) polymers on the bacterial cell surface (middle ‐ red), and an overlay of the two signals (right). b) ImageJ analysis quantitating Hoechst and Cy5 signal intensities in a cross section of one adjuvanted bacterium.

Despite the presence of approximately 57 units of CL307 per host polymer (Supporting Information: Figure S2), the polymers could still complex well to the Ad‐pre‐functionalized *S. aureus* surface. Confocal imaging showed virtually all bacteria with strong Cy5 signals indicative of complexed polymer on the bacterial cell surface, signals adjacent to Hoechst counterstaining of the cytoplasm (Figure 2a). ImageJ analysis of an adjuvanted bacterium's cross section validated these findings, with distinct Cy5 signals flanking the Hoechst signal of the bacterium's cytoplasm (Figure 2b).

**Figure 3:**
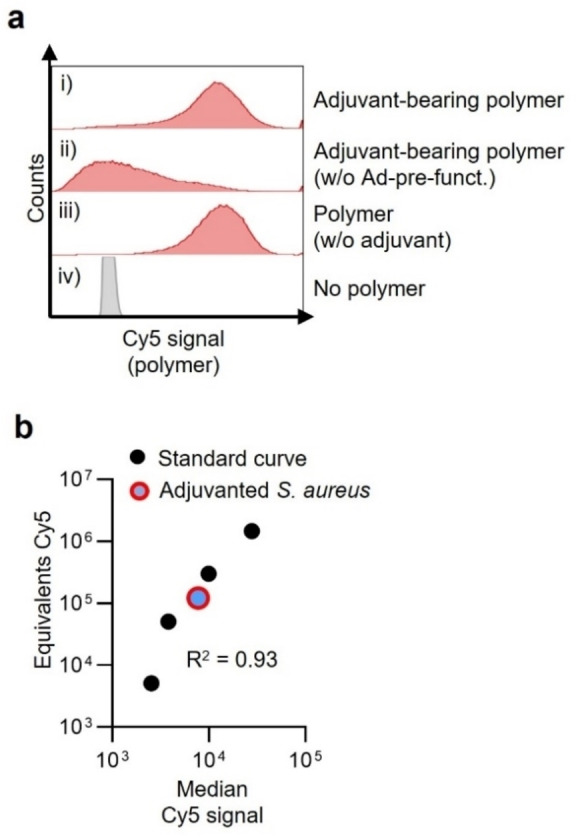
Quantitative flow cytometric analysis of adjuvanted *S. aureus*. a) Representative flow cytometry histogram of Cy5 signals (x‐axis, log scale) in a sample of *S. aureus* complexed with CL307‐bearing polymer (i), *S. aureus* complexed with little CL307‐bearing polymer in the absence of Ad‐pre‐functionalization (ii), *S. aureus* complexed with polymer lacking CL307 (iii) and control *S. aureus* (iv). b) Plot of adjuvanted *S. aureus*’ median Cy5 signal (red‐blue) in relation to fluorescence quantification beads (black) of varying Cy5 intensities.

Flow cytometric analysis was subsequently used to gauge the efficiency of generating adjuvanted *S. aureus*. The supramolecular system employed was found to be highly efficient, with virtually all bacteria showing varying quantities of CL307‐bearing polymer complexed (Figure 3a; median Cy5 signal of 8,610±1,914 A.U. (i) vs. 0.9±0.7 A.U. (iv)). Notably, CL307‐bearing polymers were much less effectively complexed to bacteria without Ad‐pre‐functionalization (Figure 3a, panel ii ‐ 7‐fold less). To estimate the average quantity of CL307 per bacterium, median Cy5 signal was converted into absolute equivalents Cy5 dye via commercial fluorescence quantification beads (Figure 3b). This conversion yielded an average of 10^5^ polymers per bacterium, indicating the presence of about 10^7^ units of CL307 per bacterium ‐ a quantity on the same order of magnitude as seen in adjuvanted nanoparticle vaccines.^[7]^

**Figure 4:**
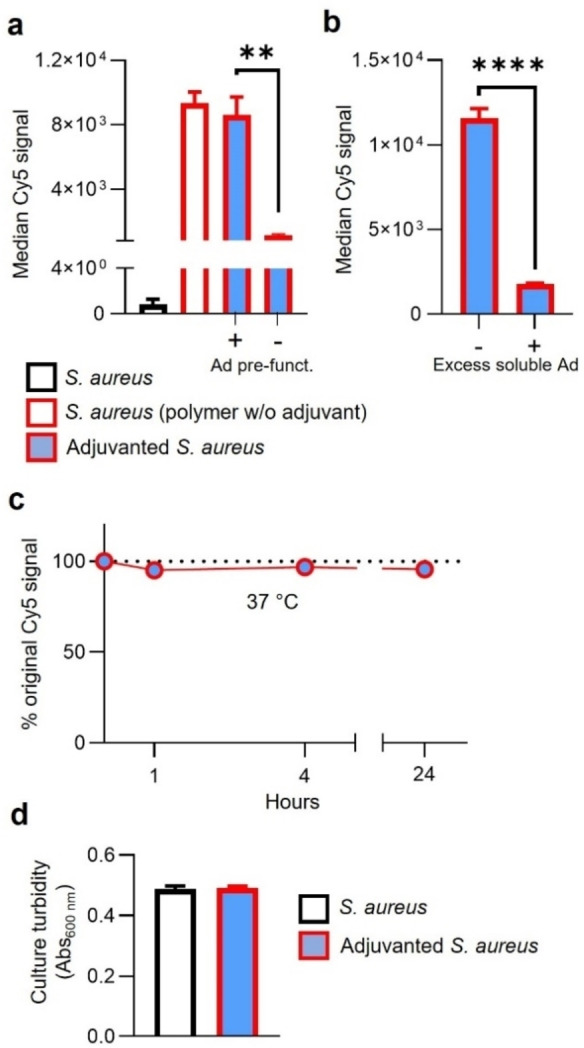
(Bio)chemical characteristics of supramolecular system underlying adjuvanted *S. aureus*. a) Median Cy5 signal (y‐axis) of flow cytometric analysis of *S. aureus* controls (black), *S. aureus* with non‐adjuvant polymer complexed (red), and adjuvanted *S. aureus* (red‐blue) in the presence (+) and absence (‐) of Ad‐pre‐functionalization (x‐axis). b) Median Cy5 signal (y‐axis) of flow cytometry‐based competition experiment of adjuvanted *S. aureus* generated in the absence and presence (x‐axis) of excess (1 mM) soluble Ad. c) Percent remaining median Cy5 signal (y‐axis) of adjuvanted *S. aureus* after 24 hours incubation (x‐axis) in PBS. d) Culture turbidity measurements by absorbance (y‐axis) of adjuvanted *S. aureus* (red‐blue) and control *S. aureus* (black) (x‐axis) after 16 hours incubation at 37 °C in growth media. All data shown are from representative experiments of n=3. Ad=adamantane; **=p<0.01, ****=p<0.0001.

Flow cytometric analysis was further used to characterize the supramolecular chemical system when adapted for introducing adjuvants onto the *S. aureus* cell surface. The presence of about 57 CL307 units per polymer did not preclude complexation at levels comparable to parental polymer analog lacking CL307 (Figure 4a; 8,610±1,914 vs. 9,342±1,171 A.U., p=0.6020). Remarkably, optimal complexation was however dependent on a slightly elevated salinity during complexation (Supporting Information: Figure S12), an effect that has previously been described for enhancing supramolecular host‐guest interactions.^[10]^ Supramolecular host‐guest interactions were critical for maximizing polymer complexation: the absence of Ad‐pre‐functionalization decreased complexation 7‐fold (Figure 4a; 1,130±122 vs. 8,610±1,914, p<0.01), and competition experiments using an excess of 1 mM soluble Ad similarly decreased complexation (Figure 4b; 1,774±74 vs. 11,574±991 A.U., p<0.0001). Once formed, adjuvanted *S. aureus* remained stably associated, with over 95 % of CL307‐bearing polymers remaining complexed after 24 hours incubation at physiological conditions (Figure 4c; 48,593±2,652 A.U. at t=24 h vs. 49,736±1,564 A.U. at t=0). In line with previous work,^[8]^ complexed CL307‐bearing polymers did not adversely affect bacterial viability, with comparable culture growth relative to control bacteria (Figure 4d; 0.492±0.005 vs. 0.486±0.012 A.U., p=0.481).

**Figure 5:**
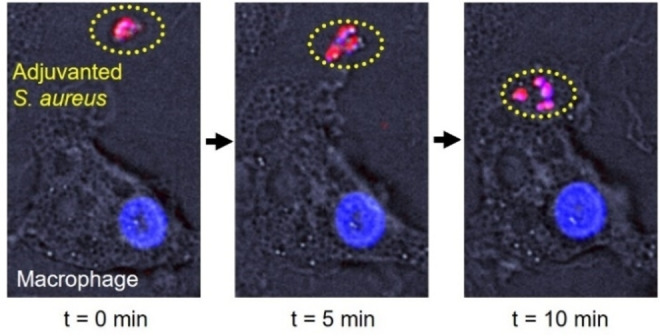
Phagocytosis of adjuvanted *S. aureus* by a monocyte‐derived macrophage. Time‐lapse confocal fluorescence microscopy images at t=0 (left), t=5 minutes (middle) and t=10 minutes (right) of a macrophage (cell nucleus in blue) encountering and phagocytosing adjuvanted *S. aureus* (yellow dotted oval).

To investigate the immunological characteristics of adjuvanted *S. aureus*, a monocyte‐derived macrophage assay was used as previously described,^[8b]^ as macrophages are reported to be key players in an effective immune responses to this pathogen.^[11]^ Confocal imaging showed that the presence of synthetic adjuvants on the *S. aureus* surface did not prevent macrophages from normally phagocytosing such bacteria (Figure 5) ‐all adjuvanted *S. aureus* were phagocytosed within 20 minutes.

**Figure 6:**
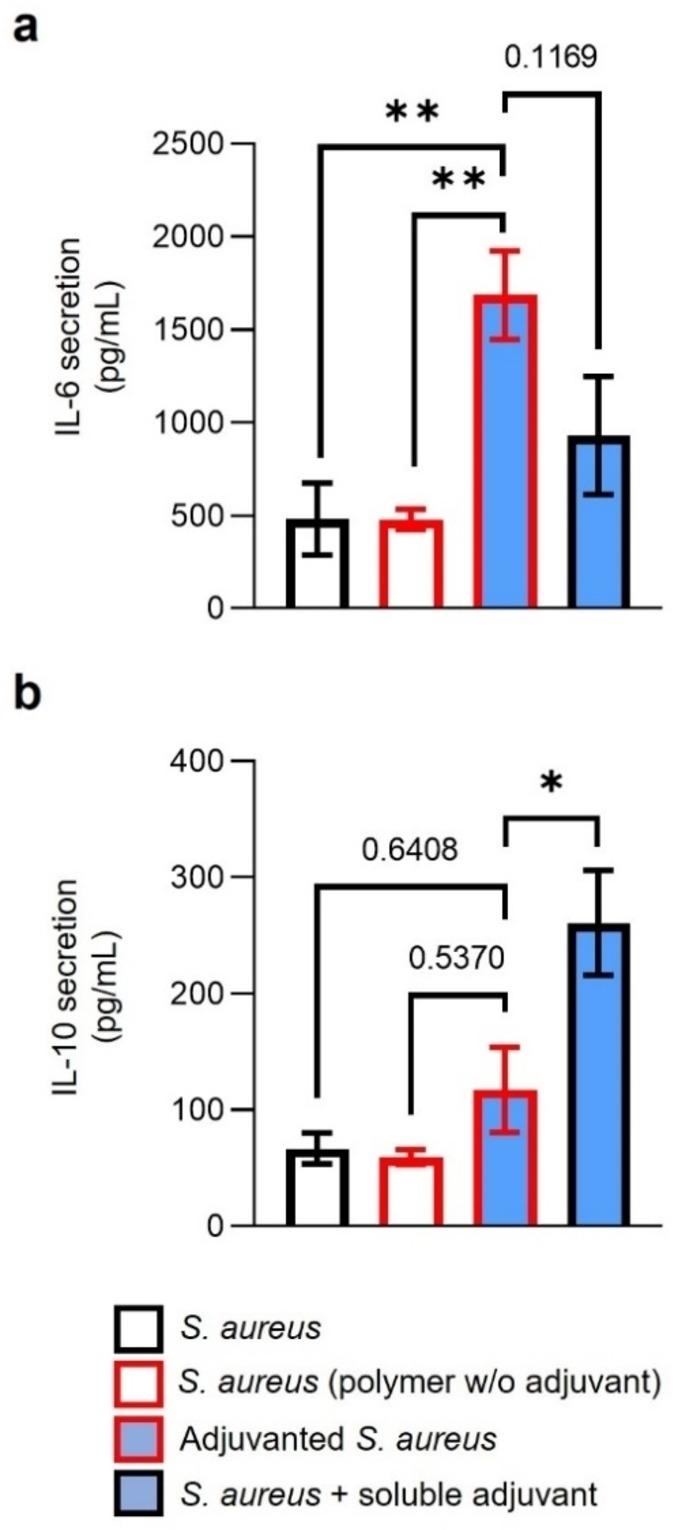
Cytokine secretion by monocyte‐derived macrophages after 24 hours of exposure to adjuvanted *S. aureus* and controls. a) IL‐6 secretion (y‐axis, pg/mL) in response to *S. aureus* controls (black), *S. aureus* bearing polymer without CL307 (red), adjuvanted *S. aureus* (red‐blue), and a mixture of *S. aureus* and soluble concentration‐matched CL307 (black‐blue) (x‐axis). b) IL‐10 secretion (y‐axis, pg/mL) in response to *S. aureus* controls (black), *S. aureus* bearing polymer without CL307(red), adjuvanted *S. aureus* (red‐blue), and a mixture of *S. aureus* and soluble concentration‐matched CL307 (black‐blue) (x‐axis). Data shown are n=5 from 2 independent experiments. IL‐6=interleukin‐6; IL‐10=interleukin‐10; *=p<0.05, **=p<0.01.

Finally, the immunogenicity of adjuvanted *S. aureus* relative to control *S. aureus* was assayed by measuring macrophage cytokine secretion during 24 hours of bacterial exposure. Adjuvanted *S. aureus* induced significantly increased secretion of pro‐inflammatory IL‐6 (Figure 6a), with 4‐fold increases compared to both control *S. aureus* (1,686±238 vs. 480±193 pg/mL, p<0.01) and *S. aureus* bearing polymer without CL307 (1,686±238 vs. 479±55 pg/mL, p<0.01). CL307 conjugated to the *S. aureus* surface also induced a trend towards a higher IL‐6 secretion compared to a mixture of *S. aureus* and soluble concentration‐matched CL307 (1,686±238 vs. 940±322 pg/mL, p=0.1169). Secretion of anti‐inflammatory IL‐10 was on the other hand less changed by adjuvanted *S. aureus* (Figure 6b). IL‐10 secretion to adjuvanted *S. aureus* was comparable to both control *S. aureus* (117±37 vs. 67±13 pg/mL, p=0.6408) and *S. aureus* bearing polymer without CL307 (117±37 vs. 60±6 pg/mL, p=0.5370). Notably, IL‐10 secretion in response to adjuvanted *S. aureus* was however significantly lower compared to *S. aureus*+CL307 mixture (117±37 vs. 261±45 pg/mL, p<0.05).

Altogether, these results indicated that macrophages responded with a slightly more pro‐inflammatory IL‐6 response to *S. aureus* whose cell surface was chemically modified with CL307 ‐ an effect that was positively impacted by physically connecting CL307 to the bacteria. Production of pro‐ and anti‐inflammatory mediators such as IL‐6 and IL‐10, respectively, are constantly balanced to properly calibrate immune responses between too much and too little activation; usually, when one goes up, so does the other. The reversed trends in cytokine production patterns of IL‐6 and IL‐10 observed for the conjugated and soluble compound (Figure 6) exemplify a shift in that balance. Whereas soluble presentation of CL307 induced a mixed pro‐ and anti‐inflammatory response in macrophages, conjugated CL307 induced a predominantly pro‐inflammatory response. Future studies will need to determine if similar shifts towards a more pro‐inflammatory response also apply to other immune cell models. Additionally, *in vivo* models will need to be used to gauge the potential (clinical) utility of the shifts here observed *in vitro*.

The findings here described suggest that chemical augmentation of microbial cell surfaces with adjuvants may indeed offer a strategy for improving such microbes’ immunogenicity. In the future, UBI_29–41_ vectors’ ability to “tag” offending bacteria *in vivo* ^[12]^ could provide a basis to exploit supramolecular pre‐targeting strategies^[13]^ that locally introduce immunogenic moieties. Introduction thereof could then help activate the immune system and promote clearance of the infectious agent. Another potential application of the presented concept is in the field of vaccinology. Despite the challenging regulatory complexity of bringing live attenuated vaccines to the clinic, there nevertheless exist several ‐ most notably, against tuberculosis and malaria ‐ that continue to be the subject of active investigation.^[14]^ As these vaccines are still characterized by suboptimal performance attributed to a low immunogenicity,^[15]^ chemical modification could help provide a much needed boost in immunogenicity.

## Conclusion

In conclusion, we have here shown that supramolecular introduction of a TLR7 agonist‐based adjuvant (CL307) onto poorly immunogenic *S. aureus* bacteria yields increased production of the key pro‐inflammatory cytokine IL‐6. Future work will need to determine whether a similarly improved immune response is more broadly observed for other players of the immune system in the context of antimicrobial responses, such as neutrophils, and whether such improvements ultimately help the immune system more effectively clear poorly immunogenic pathogens. A key area of research to this end will be investigating how the presented concept translates to an *in vivo* setting. Eventually, positive findings could open up the possibility of using chemical functionalization of (microbial) cells to fine‐tune immunological properties, and in this way rationally guide effective immune responses.

## Experimental Section

Full procedures related to synthesis and analysis of the chemical compounds used can be found in the Supporting Information.

**Synthesis of supramolecular components**: To create the Ad‐UBI_29–41_ compound for pre‐functionalizing the bacterial cell surface with supramolecular guest, a UBI_29–41_ peptide (H‐TGRAKRRMQYNRR‐NH_2_) was synthesized in‐house by automated Fmoc‐based SPPS. Subsequently, Fmoc‐Lys(Fmoc)‐OH was coupled manually to the liberated N‐terminus of the resin‐bound peptide using PyBOP and DiPEA in DMF. After Fmoc deprotection of the lysine sidechain and N‐terminus, Fmoc‐Gly‐OH was coupled using PyBOP and DiPEA by agitating in DMF. The glycine amines were liberated and, finally, 1‐adamantanecarbonyl chloride was coupled using 1‐hydroxybenzotriazole and DiPEA by agitating in DMF. The peptide was then cleaved by adding a mixture of v/v 38 : 1 : 1 TFA:TIPS:H_2_O and agitating for 3 hours, whereafter the mixture was added to ice‐cold MTBE : hexane 1 : 1. The crude peptide was collected by centrifugation, whereafter the pellet was resuspended in ice‐cold MTBE : hexane twice, followed by drying *in vacuo*. The off‐white solid was then purified using preparative HPLC, followed by lyophilization to yield the white solid product.

To create the adjuvant‐bearing host polymer CD_85_Cy5_2_CL307_58_PIBMA for complexing onto Ad‐pre‐functionalized bacterial cell surface, poly(isobutylene‐alt‐maleic‐anhydride was dissolved in dimethylsulfoxide, whereafter amino(6‐monodeoxy‐6‐mono)‐β‐cyclodextrin hydrochloride and N,N‐Diisopropylethylamine were added, followed by stirring at 80 °C for 94 hours. The solution was purified by dialysis in water for 7 hours, followed by dialysis in phosphate buffer (0.2 M, pH 9) for 144 hours including refreshment of buffer twice, followed by dialysis in water for 7 hours. The dialysate was discarded and the residue was lyophilized, yielding an off‐white solid. The product PIBMA_[389]_‐CD_[85]_ was subsequently dissolved in water, whereafter *N*,*N*′‐diisopropylcarbodiimide was added. The mixture was stirred at room temperature for 1 hour followed by addition of NH_2_‐Cy5‐COOH. The solution was stirred for 5 hours at room temperature whereafter it was dialyzed in water for 24 hours with one refresh of water, followed by lyophilization of the product. The product PIBMA_[389]_‐CD_[85]_‐Cy5_[2]_ was dissolved in water, followed by addition of DIC. After stirring for 1.3 hours at room temperature, CL307 was added. After shaking for 1.3 hours, ethanolamine was added and stirring was continued for another 16 hours at room temperature. Thereafter, the reaction mixture was dialyzed in water for 29 hours with one refreshment of water. The resulting adjuvant‐bearing host polymer CD_85_Cy5_2_CL307_58_PIBMA was used as is for experiments; PBS pH 7.4 was added where necessary.

**Supramolecular complexation of CL307‐bearing polymers onto** ***S. aureus*** **cell surface**: *S. aureus* stocks (strain ATCC 29213) were thawed from −20 °C and washed twice in 25 mM ammonium acetate buffer pH 5 by centrifugation at 10k RCF for 5 minutes and subsequent removal of supernatant by pipet. From this washed stock, 10^8^ bacteria were added to 1 mL of an 8 μM solution of Ad‐UBI_29–41_ in 25 mM ammonium acetate buffer pH 5 and incubated for 30 minutes at 37 °C with shaking. Thereafter, bacteria were thrice washed with PBS containing 1 % albumin by centrifugation at 10k RCF for 5 minutes and subsequent removal of supernatant by decanting. Washed, Ad‐pre‐functionalized bacteria were resuspended in 1 mL PBS, wherefrom 100 μL aliquots were added to 100 μL of a 2 μM solution of the adjuvant‐bearing host polymer (CD_85_Cy5_2_CL307_57_PIBMA_389_) in PBS at a concentration of 1.9X (where 1X is equivalent to standard commercial PBS) and incubated overnight at 37 °C with shaking. Thereafter, bacteria were thrice washed with PBS containing 1 % albumin by centrifugation at 10k RCF for 5 minutes and subsequent removal of supernatant by decanting. Washed, adjuvanted bacteria were then resuspended in PBS or RPMI for downstream applications.

**Analysis of adjuvanted** ***S. aureus***: To visually confirm the presence of CL307‐bearing host polymers on the *S. aureus* surface via the polymers’ Cy5 reporter, 10 μL aliquots (with the addition of 10 μg/mL Hoechst 33342) of adjuvanted bacteria were added to glass‐bottom confocal dishes (MatTek Corporation, Ashland, MA, USA), overlaid with a coverslip and analyzed with a Leica (Wetzlar, Germany) SP8 confocal fluorescence microscope; Hoechst signal was acquired using a 405 nm laser at 20 % maximal output and a HyD detector set to 425–475 nm, Cy5 signal was acquired using a 633 nm laser at 5 % maximal output and a HyD detector set to 650–700 nm. Post‐acquisition analyses of microscopic images was performed using Leica LasX and ImageJ software.

To quantitatively assay the presence of CL307‐bearing host polymers on the *S. aureus* surface, adjuvanted bacteria were run through a BD (Franklin Lakes, NJ, USA) FACSCanto II instrument using signal acquisition in the instrument's “APC” channel to provide a quantitative measure of CL307‐bearing host polymers present on the bacterial surface. Alongside adjuvanted bacteria, commercial fluorescence quantification beads (Bangs Laboratories, Inc., Fishers, IN, USA) were run on the instrument to generate a standard curve for converting the instrument's fluorescent signal intensities of arbitrary values to absolute values. Post‐acquisition analyses was performed with FlowJo 10 software (Ashland, OR, USA).

For assessing stability of Cl307‐polymers complexed to the *S. aureus* surface, adjuvanted bacteria were incubated at 37 °C in PBS for up to 24 hours. For assessing their growth, bacteria were incubated at 37 °C in brain heart infusion growth media with shaking for 16 hours.

**Immune response to adjuvanted** ***S. aureus***: Monocyte‐derived macrophages were prepared as previously described.^[16]^ To visually confirm that adjuvanted *S. aureus* could still be phagocytosed by macrophages, harvested macrophages were plated at a density of 100k macrophages in 200 μL RPMI containing 10 % FBS onto glass‐bottom confocal dishes and incubated overnight at 37 °C. The next day, 1.8 mL RPMI 10 % FBS containing 10 μg/mL Hoechst 33342 was added to the confocal dishes and the dishes positioned on an Andor (Belfast, Northern Ireland) Dragonfly 500 spinning disk confocal fluorescence microscope. Immediately before image acquisition, a 10 μL aliquot containing 10^6^ adjuvanted bacteria was carefully added just above the macrophage layer. Subsequently, z‐stack images of approximately 40 μm in depth at 1 μm intervals were acquired every 2.5 minutes for a total duration of 30 minutes. Post‐acquisition analyses of images were performed using Imaris software (Zurich, Switzerland).

To assay the immunogenicity of adjuvanted *S. aureus*, macrophages post‐harvesting were plated at a density of 100k macrophages in 100 μL RPMI 10 % FBS in 96‐well flat‐bottom plates (Nunc, Thermo Fisher Scientific, Waltham, MA, USA) and incubated overnight at 37 °C. The next day, 400k adjuvanted bacteria in 100 μL RPMI 10 % FBS were added to the wells, and the macrophages incubated for another 24 hours. Following this, supernatants were removed from wells and stored at −20 °C for eventual cytokine analysis using BD Biosciences human IL‐6 (Cat. No. 555220) and IL‐10 (Cat. No. 555157) ELISA kits according to the manufacturer's specifications.

**Statistical analyses**: Significance of differences between groups was analyzed by ANOVA with t‐test for multiple comparison's or Student's t‐test for two groups, using Graphpad Prism 9.3.1.

## Conflict of interest

The authors declare no conflict of interest.

1

## Supporting information

As a service to our authors and readers, this journal provides supporting information supplied by the authors. Such materials are peer reviewed and may be re‐organized for online delivery, but are not copy‐edited or typeset. Technical support issues arising from supporting information (other than missing files) should be addressed to the authors.

Supporting Information

## Data Availability

The data that support the findings of this study are available on request from the corresponding author. The data are not publicly available due to privacy or ethical restrictions.
