# Supplementary material for: Chemically Enhanced Immunogenicity of Bacteria by Supramolecular Functionalization with an Adjuvant
Source: Chembiochem. 2022 Nov 4;23(23):e202200434. doi: 10.1002/cbic.202200434 (PMC10098600; doi:10.1002/cbic.202200434)
Supplement: Supplementary file 1 — Supporting Information [file CBIC-23-0-s001.pdf]

ChemBioChem

Supporting Information

Chemically Enhanced Immunogenicity of Bacteria by Supramolecular Functionalization with an Adjuvant

Nikolas Duszenko, Danny M. van Willigen, Anton Bunschoten, Aldrik H. Velders, Meta Roestenberg, and Fijs W. B. van Leeuwen*

Table of Contents

A. Chemical Procedures

1. General
   1.1. Abbreviations
   1.2. Chemicals
   1.3. HPLC
   1.4. Mass spectrometry
   1.5. NMR
   1.6. Dialysis
   1.7. Photometry
2. Syntheses
   2.1. UBI-Ad2 (1)
   2.2. Indole-COOH (2)
   2.3. Indole-Phth (3)
   2.4. Phth-Cy5-COOH (4)
   2.5. NH_2-Cy5-COOH (5)
   2.6. PIBMA[389]-CD[85] (6)
   2.7. PIBMA[389]-CD[85]-Cy5[2] (7)
   2.8. PIBMA[389]-CD[85]-Cy5[2]-NEtOH[691] (8)
   2.9. PIBMA[389]-CD[85]-Cy5[2]-CL307[58]-NEtOH[633] (9)
3. Chemical analyses
   3.1. Quantification of polymer-bound β-CD
   3.2. Quantification of polymer-bound NH2-Cy5-COOH
   3.3. Quantification of polymer-bound CL307
   3.4. NMR
   3.5. HPLC

B. Additional Figures

Chemical Procedures

1. General

1.1 Abbreviations

Ac_2O: acetic anhydride

Ad: adamantane

CD: β-cyclodextrin

CL307: the adjuvant, a TL7 agonist

COSY: correlated spectroscopy

D_2O: deuterium oxide

DCM: dichloromethane

DIC: N,N'-diisopropylcarbodiimide

DiPEA: N,N'-diisopropylethylamine

DMF: dimethylformamide

DMSO: dimethylsulfoxide

EtOH: ethanol

Fmoc: 9-fluorenylmethoxycarbonyl

MeOD: deuterated methanol

MeOH: methanol

MTBE: methyl tert-butyl ether

NEtOH: ethanolamine

NMR: nuclear magnetic resonance

Phth: phthalimide

PyBOB: benzotriazol-1-yloxytripyrrolidinophosphonium hexafluorophosphate

SPPS: solid-phase peptide synthesis

TFA: Trifluoroacetic acid

TIPS: triisopropyl silane

UBI: ubiquicidin

1.2 Chemicals

Chemicals were obtained commercially from Merck (Darmstadt, Germany), TCI (Tokyo, Japan) or Cyclodextrin-Shop (Tilburg, The Netherlands) and used without further purification; these were deemed free of any significant endotoxin contamination by respective manufacturers. Amino acids were obtained from either Bachem (Bubendorf, Switzerland) or Iris Biotech (Marktredwitz, Germany). Solvents were obtained from Actu-All (Oss, The Netherlands), Biosolve (Valkenswaard, The Netherlands) or Merck (Darmstadt, Germany). Acetonitrile, N,N-Dimethylformamide and Dimethylsulfoxide were dried using 4Å molecular sieves Merck (Darmstadt, Germany) unless stated otherwise.

Reactions were carried out under normal atmosphere unless stated otherwise. Column chromatography was performed with 40–63 µm silica from Screening Devices (Amersfoort, The Netherlands). SPPS was carried out either by a Biotage Syro II (Uppsala, Sweden) or by hand using fritted tubes (6, 10 or 25 mL) from Screening Devices (Amersfoort, The Netherlands) and in-house N_2 flow/vacuum.

1.3 HPLC

High-performance liquid chromatography was performed on a Waters HPLC system using either a 1525EF or 2545 pump and a 2489 UV/VIS detector. For preparative HPLC either a Dr. Maisch GmbH Reprosil-Pur 120 C18-AQ 10 µm (250 × 20 mm) column using a flow of 12 mL/min or an XBridge Prep C8 10 µm OBD (250 × 30 mm column) with a flow of 25 mL/min was used. For semi-preparative HPLC a Dr. Maisch GmbH Reprosil-Pur C18-AQ 10 µm (250 × 10 mm) column was used with a flow of 5 mL/min. For analytical HPLC a Dr. Maisch GmbH Reprosil-Pur C18-AQ 5 µm (250 × 4.6 mm) column with a flow of 1 mL/min and a gradient of 5→95% CH_3CN in H_2O) (0.1% TFA) in 40 min (1 mL/min) was used.

1.4 Mass spectrometry

Mass spectrometry was performed using a Bruker Microflex Matrix-assisted laser desorption ionization time-of-flight (MALDI-TOF) mass spectrometer (Billerica, MA, United States).

1.5 NMR

^{1}H NMR, COSY and ^{13}C NMR of the dyes were recorded on a Bruker AV-300 spectrometer (300 MHz) (Billerica, MA, United States) in methanol-d_4. Quantification of the number of β-CD units per polymer with ^{1}H NMR and DOSY was done in D_2O using a Bruker Avance III spectrometer (500 MHz), equipped with a 5 mm TXI probe.

1.6 Dialysis

Dialysis was performed using Pur-A-Lyzer (either Mega/Maxi 3500 MWCO or Mini 12000 MWCO) dialysis kits from (Sigma-Aldrich, St. Louis, MO, USA).

1.7 Photometry

Absorbance spectra were recorded using an Ultrospec 2100 pro (Amersham Biosciences, Little Chalfont, United Kingdom).

2. Syntheses

2.1 UBI-Lys(Gly-Ad)$_2$ (1)

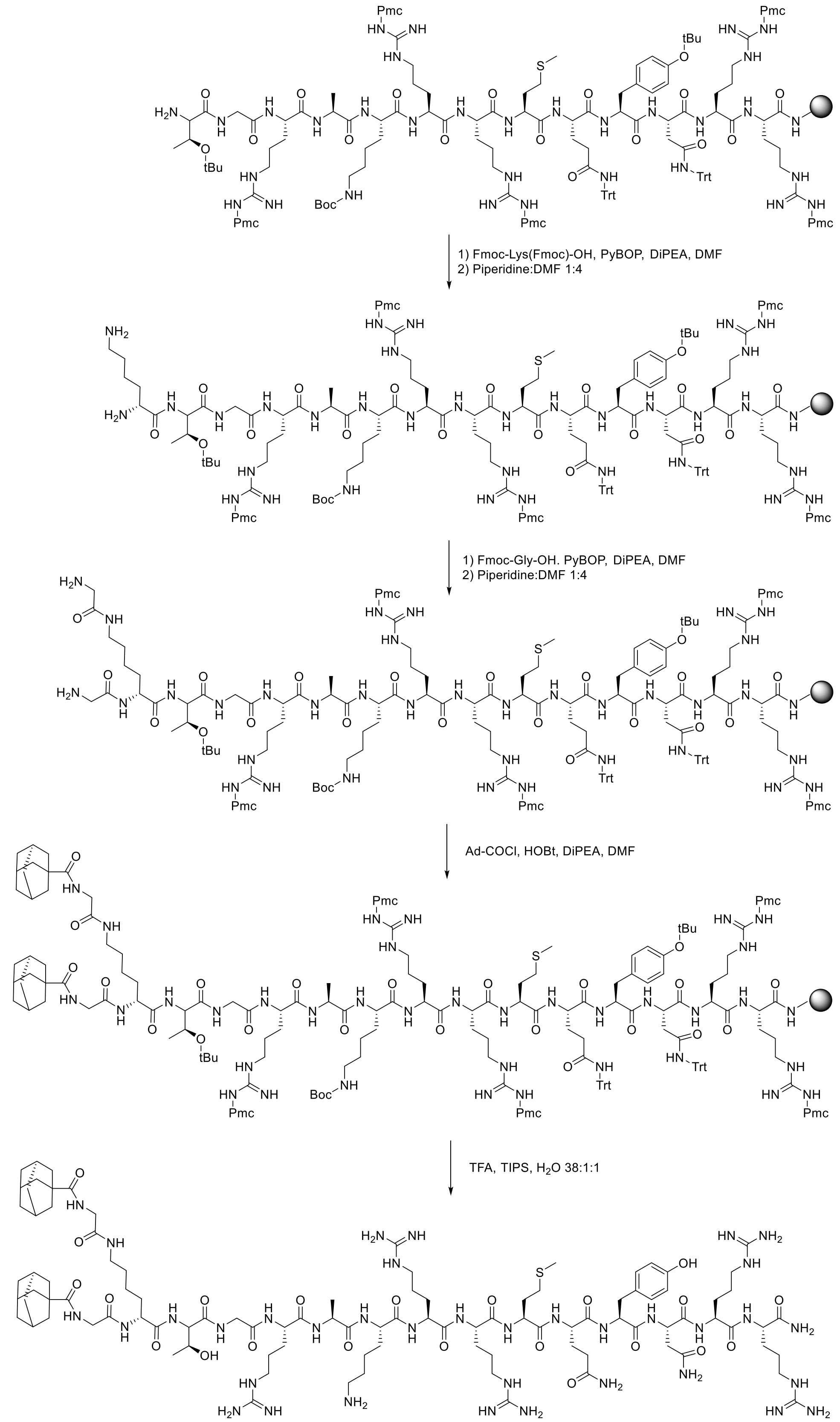

The $UBI_{29\text{-}41}$ peptide (H-TGRAKRRMQYNRR-NH_2) was synthesized in-house on a 10.0 µmol scale using Tentagel SRAM resin (Rapp Polymere GmbH) by automated Fmoc-based SPPS. Subsequently, Fmoc-Lys(Fmoc)-OH (12.0 mg, 20.1 µmol) was coupled manually to the liberated N-terminus of the resin-bound peptide using PyBOP (21.0 mg, 40.2 µmol) and DiPEA (26.2 µL, 150.7 µmol) in DMF (3.0 mL) by agitating for 4 hours at room temperature. After Fmoc deprotection of the lysine sidechain and N-terminus, Fmoc-Gly-OH (12.0 mg, 40.2 µmol) was coupled (using PyBOP (21.0 mg, 40.2 µmol) and DiPEA (26.2 µL, 150.7 µmol) by agitating in DMF (3.0 mL) at room temperature for 18 hours. The glycine amines were liberated and, finally, 1-Adamantanecarbonyl chloride (8.0 mg, 150.7 µmol) was coupled using 1-hydroxybenzotriazole (6.0 mg, 40.2 µmol) and DiPEA (26.2 µL, 150.7 µmol) by agitating in DMF (3.0 mL) for 22 hours at room temperature. The peptide was then cleaved by adding a mixture of v/v 38:1:1 TFA:TIPS:H_2O (2 mL) and agitating for 3 hours at room temperature, whereafter the mixture was added to ice-cold MTBE:Hexane 1:1 (200.0 mL). The crude peptide was collected by centrifugation, whereafter the pellet was resuspended in ice-cold MTBE:Hexane twice, followed by drying *in vacuo*. The off-white solid was then purified using preparative HPLC (5→95% acetonitrile in 40 minutes) followed by lyophilization, yielding a white solid (6.4 mg, 28.2 % overall yield).

MALDI-TOF (Positive mode): Calculated for $C_{100}H_{168}N_{36}O_{22}S$: 2258.3, found 2258.2.

2.2 Indole-COOH (2)

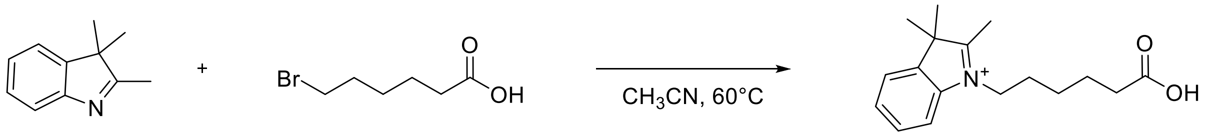

6-bromohexanoic acid (2.5 g, 12.6 mmol) was dissolved in dry acetonitrile (10.0 mL) followed by addition of 2,3,3-trimethylindolenine (1.0 mL, 6.3 mmol). The brown solution was stirred at 60°C for 60 hours whereupon it turned into a dark red solution. Acetonitrile was removed by vacuum and after dissolving the remaining solid in MeOH the product was precipitated in ethyl acetate. Filtration over a sintered glass filter (P3) followed by washing with ethyl acetate and drying *in vacuo* yielded a light pink solid (447.0 mg, 25.8 % isolated yield) which was used without further purification.

2.3 Indole-Phth (3)

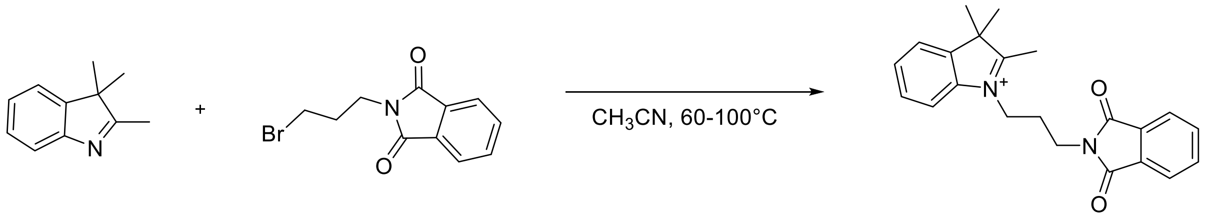

2,3,3-trimethylindolenine (504.0 µL, 3.2 mmol) was dissolved in dry acetonitrile (5.0 mL) and N-(3-bromopropyl)phthalimide (843.0 mg, 3.2 mmol) was added. The mixture was heated to 100°C for 4 hours, followed by 96 hours at 60°C. A red solid had formed and the mixture was allowed to cool, whereafter acetonitrile was decanted. The solid was dissolved in acetone and the product was precipitated in diethyl ether. The pink supernatant was decanted and the slightly orange solid was redissolved and reprecipitated in diethyl ether; this process was repeated until the supernatant was colorless. The orange precipitate was then filtrated using a sintered glass filter (P3) and dried *in vacuo* resulting in an orange solid (1.2 g, 89.6% isolated yield) which was used without further purification.

2.4 Phth-Cy5-COOH (4)

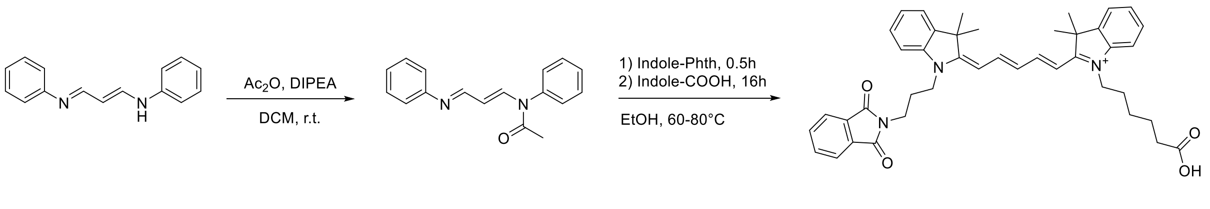

Malonaldehyde dianilide hydrochloride (100.0 mg, 0.4 mmol) was activated by acetylation as described by Oushiki et al.[1] After evaporation of dichloromethane it was redissolved in ethanol and **Indole-Phth (3)** (162.0 mg, 0.4 mmol) was added, resulting in a red solution which turned purple after 30 minutes of stirring at 60°C. **Indole-COOH (2)** (104.0 mg,

0.4 mmol) and pyridine (152.0 µL, 1.9 mmol) were added and the mixture was refluxed overnight. After cooling down, the solvents were evaporated and the residue was dissolved in acetone followed by precipitation in diethyl ether. The suspension was poured onto a sintered glass filter (P3) followed by washing with diethyl ether and drying *in vacuo*. The solid was then dissolved in 1:4 methanol/ethyl acetate and purified by silica column chromatography using a gradient of methanol:ethyl acetate 1:4→1:1. The fractions containing product were combined and the solvent was removed *in vacuo*, resulting in a blue solid (84.2 mg, 33.9% isolated yield).

^{1}H NMR (300 MHz, MeOD): δ 8.23 (dd, *J* = 23.6, 12.8 Hz, 1H), 7.89 – 7.76 (m, 4H), 7.52 – 7.19 (m, 8H), 6.49 (t, *J* = 12.4 Hz, 1H), 6.24 (dd, *J* = 17.0, 13.8 Hz, 2H), 4.23 – 4.09 (m, 4H), 3.83 (t, *J* = 7.2 Hz, 2H), 2.33 (t, *J* = 7.2 Hz, 2H), 2.28 – 2.16 (m, 2H), 1.91 – 1.77 (m, *J* = 15.0, 7.7 Hz, 2H), 1.72 (d, J = 2.6 Hz, 12H), 1.58 – 1.45 (m, 2H), 1.22 (s, 2H).

^{13}C NMR (75 MHz, MeOD): δ 175.80, 173.92, 172.75, 168.35, 154.48, 153.85, 142.05, 142.02, 141.38, 141.03, 134.04, 131.94, 128.40, 128.32, 125.33, 125.12, 124.69, 122.86, 122.04, 110.85, 110.29, 103.38, 102.60, 49.34, 49.05, 43.53, 41.14, 35.06, 33.18, 29.72, 26.82, 26.55, 26.45, 25.94, 24.28.

MALDI-TOF (CHCA, Positive mode): Calculated for $C_{42}H_{46}N_3O_4^+$: 656.8, found 656.8.

2.5 NH$_2$-Cy5-COOH (5)

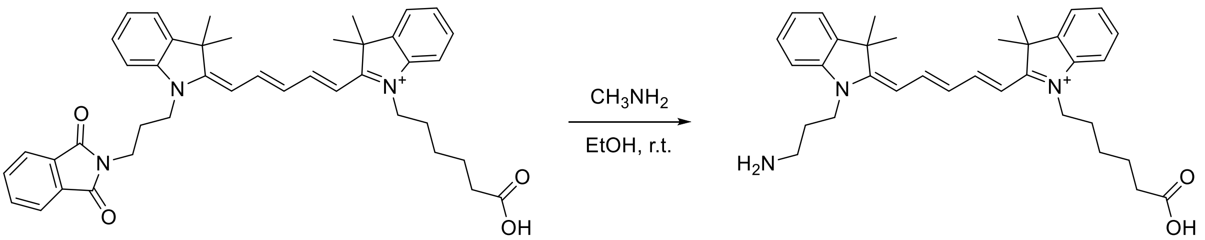

The amine was deprotected using a protocol from literature[2]. 20.0 mL of 33 wt. % methylamine in ethanol was added to **Phth-Cy5-COOH (4)** (44.0 mg, 67.0 µmol), and the colorless solution was stirred for 2 hours at room temperature. The solvents were then removed by evaporation, resulting in a blue solid which was redissolved in 2.0 mL of acetonitrile containing 0.1% TFA, followed by addition of 2.0 mL of water containing 0.1% TFA. Purification by semi-preparative HPLC yielded a blue solid after lyophilization (23.2 mg, 65.8% isolated yield).

^{1}H NMR (300 MHz, MeOD): δ 8.33 – 8.14 (m, 2H), 7.52 – 7.15 (m, 8H), 6.60 (t, J = 12.4 Hz, 1H), 6.27 (dd, J = 26.9, 13.7 Hz, 2H), 4.13 (dd, *J* = 14.2, 6.9 Hz, 4H), 3.07 (dd, *J* = 8.9, 7.0 Hz, 2H), 2.28 (t, *J* = 7.2 Hz, 2H), 2.16 – 2.04 (m, *J* = 15.0, 7.6 Hz, 2H), 1.86 – 1.74 (m, 2H), 1.70 (s, 12H), 1.52 – 1.39 (m, *J* = 15.6, 9.8, 6.0 Hz, 2H), 1.18 (s, 4H).

^{13}C NMR (75 MHz, MeOD): δ 175.80, 174.69, 172.16, 154.99, 153.70, 141.96, 141.90, 141.53, 140.89, 128.46, 128.36, 125.50, 125.46, 124.54, 122.13, 122.09, 111.14, 109.87, 103.97, 102.04, 49.57, 48.88, 43.67, 40.25, 36.77, 33.17, 29.71, 26.87, 26.66, 26.35, 25.90, 24.99, 24.25.

MALDI-TOF (CHCA, Positive mode): Calculated for $C_{34}H_{44}N_3O_2^+$: 526.7, found 527.1.

2.6 PIBMA[389]-CD[85] (6)

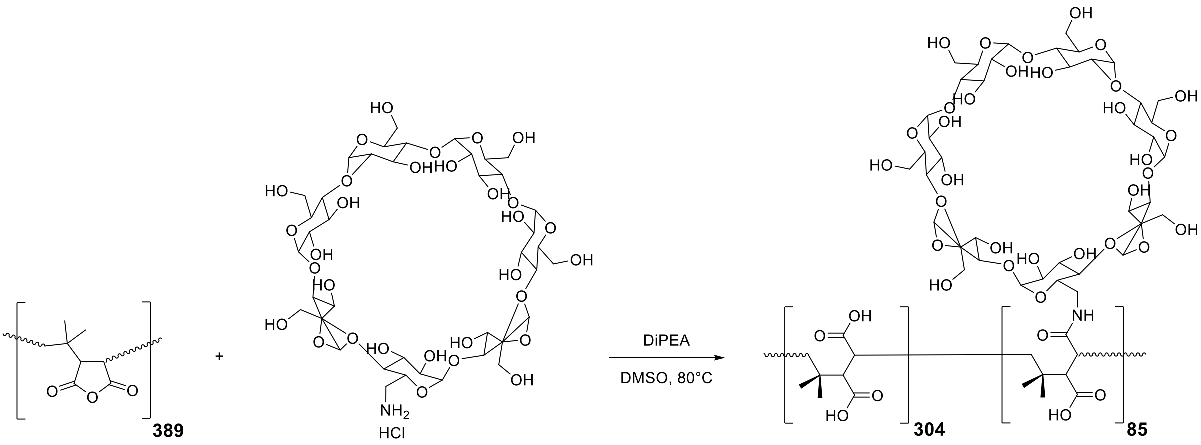

This compound was synthesized based on a previously published procedure.[3] Poly(isobutylene-alt-maleic-anhydride (200.0 mg, 3.3 µmol) was dissolved in dimethylsulfoxide (3.0 mL), whereafter Amino(6-monodeoxy-6-mono)-β-cyclodextrin hydrochloride (620.3 mg, 530.0 µmol) and N,N-Diisopropylethylamine (29.0 µL, 166.7 µmol) were added, and stirring at 80°C was carried out for 94 hours. The solution was purified by dialysis in water (1000.0 mL) for 7 hours, followed by dialysis in phosphate buffer (0.2 M, pH 9, 1000.0 mL) for 144 hours including refreshment of buffer twice, followed by dialysis in water (1000.0 mL) for 7 hours. The dialysate was discarded and the residue was lyophilized, yielding an off-white solid (453.6 mg, 85.1% isolated yield).

2.7 PIBMA[389]-CD[85]-Cy5[2] (7)

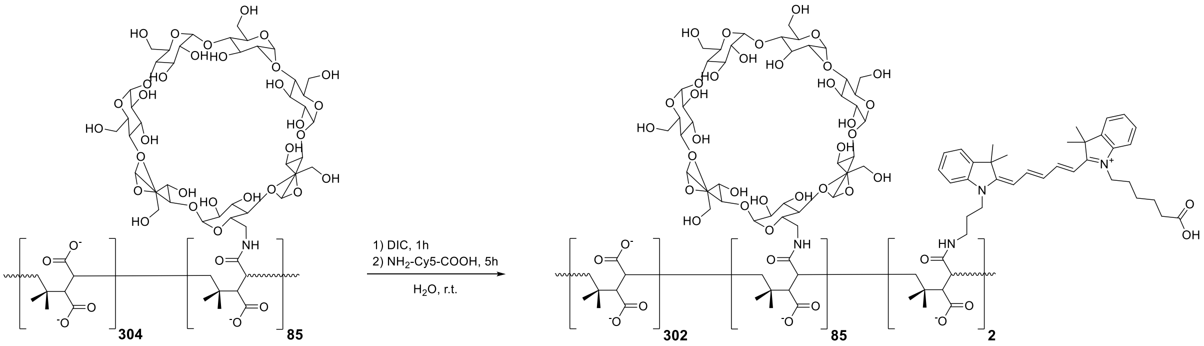

This compound was synthesized using a coupling method published by Fattahi et al.[4] **PIBMA[389]-CD[85] (6)** (420.0 mg, 2.6 µmol) was dissolved in water, whereafter N,N'-Diisopropylcarbodiimide (122.8 µL, 798.0 µmol) was added. The mixture was stirred at room temperature for 1 hour followed by addition of 2.4 mL of a 1.1 mg/mL solution of **NH_2-Cy5-COOH (5)** in 1:8 ethanol/water (2.8 mg, 5.3 µmol). The solution was stirred for 5 hours at room temperature whereafter it was dialysed in water (5000.0 mL) for 24 hours while refreshing the water once, followed by lyophilization of the residue.

2.8 PIBMA[389]-CD[85]-Cy5[2]-NEtOH[691] (8)

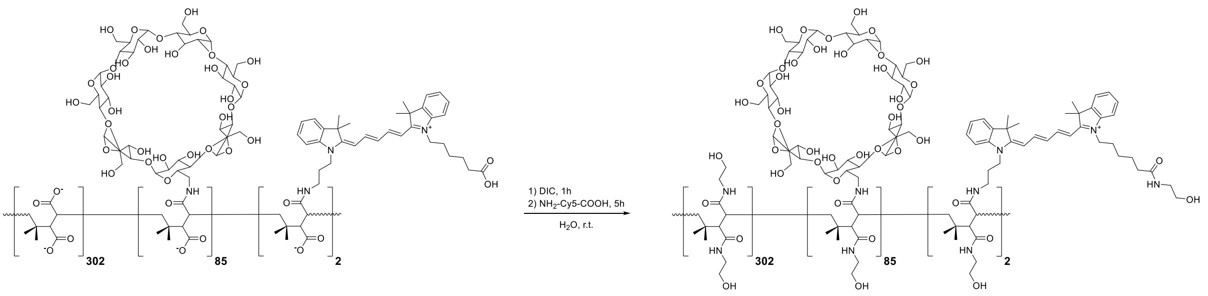

This compound was synthesized by dissolving **PIBMA$_{[389]}$-CD$_{[85]}$-Cy5$_{[2]}$ (7)** (1.0 mg, 6.3 nmol) in water (200.0 µL) and adding DIC (2.4 µL, 1.9 µmol). After stirring at room temperature for 1 hour, ethanolamine (1.0 µL, 16.3 µmol) was added and stirring was continued for 5 hours, followed by dialysis in water (5000.0 mL) for 48 hours with one refreshment of water. The residue was used as is for experiments; PBS pH 7.4 was added where necessary.

2.9 PIBMA$_{[389]}$-CD$_{[85]}$-Cy5$_{[2]}$-CL307$_{[58]}$-NEtOH$_{[633]}$ (9)

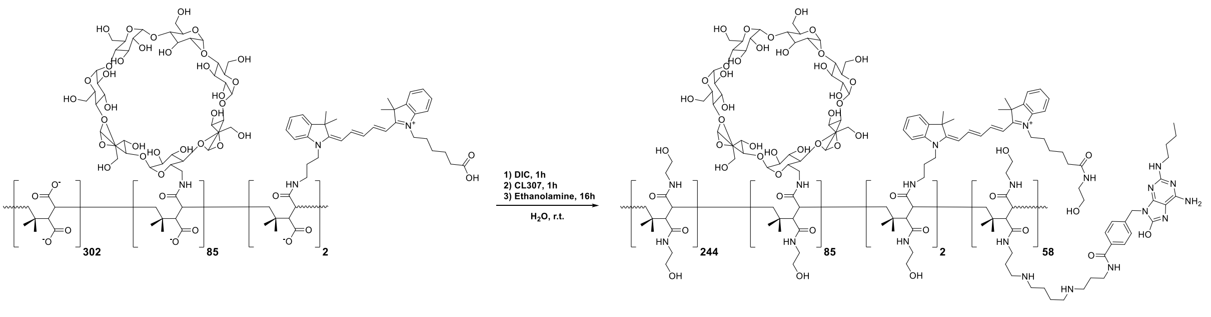

This compound was synthesized by dissolving **PIBMA$_{[389]}$-CD$_{[85]}$-Cy5$_{[2]}$ (7)** (8.05 mg, 50.3 nmol) in water (805 µL), followed by addition of DIC (7.7 µL, 50.3 µmol). After stirring for 1.3 hours at room temperature, CL307 (1.5 mg, 2.5 µmol) in water (1.5 mL) was added. After shaking for 1.3 hours ethanolamine (9.1 µL, 150.9 µmol) was added and stirring was continued for another 16 hours at room temperature. Thereafter, the reaction mixture was dialyzed in water (5 L) for 29 hours with one refreshment of water. The residue was used as is for experiments; PBS pH 7.4 was added where necessary.

3. Chemical analyses

3.1 Quantification of β-CD

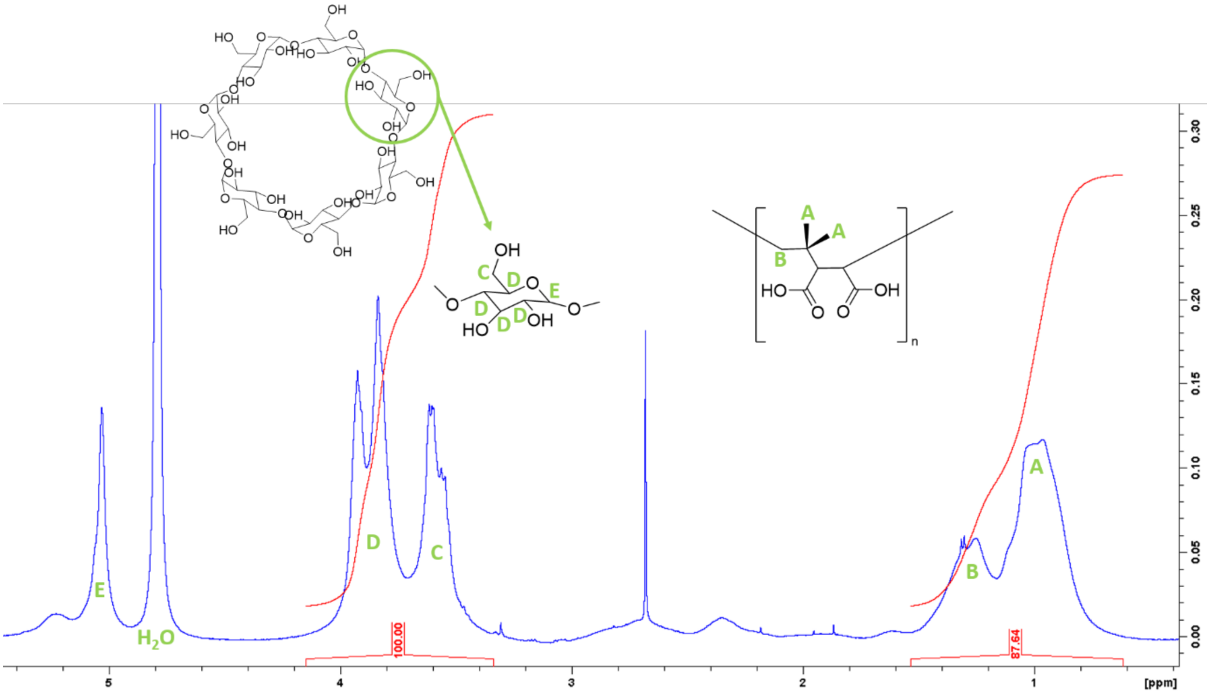

Figure S1: ^{1}H NMR spectrum with integrated peaks and annotation of *PIBMA$_{[389]}$-CD$_{[85]}$ (6)* confirming presence of β-CD, which was used for calculation of the average amount of β-CD per polymer unit.

The amount of β-CD per polymer was determined as previously described[3, 5] using ^{1}H NMR; briefly: the polymer peaks at 0.8 - 1.4 ppm (CH_3 and CH_2) were integrated (87.6), as well as the β-CD peaks at 4.0 - 3.5 ppm originating from the β-CD protons. These integrals were used for calculation of the amount of β-CD per polymer using the following rationale:

$$Monomer\ protons : \beta CD\ protons = 87.6 : 100 = 8 : 9.13$$

$$\beta CD\ per\ monomer = \frac{Integrated\ \beta CD\ protons}{Total\ protons\ in\ \beta CD} = \frac{9.13}{42} = 0.217\ \beta CD = 1\ \beta CD\ per\ 4.6\ monomers$$

$$\beta CD\ units\ per\ polymer\ unit = \frac{Monomer\ units\ per\ polymer}{Monomer\ units\ per\ \beta CD} = \frac{389}{4.6} = 84.6$$

This was rounded to 85 for clarity of illustration and calculation.

3.2 Quantification of Cy5

The molar extinction coefficient of NH_2-Cy5-COOH was determined as previously described;[2] briefly: a weighed amount of Cy5 was dissolved in water to create a 5 mM stock solution. From this stock a dilution range from 7.5 µm to 0.25 µm was made in triplicate, of which the absorption was measured at Abs_{max} = 640 nm. Using linear regression, the molar extinction coefficient was then determined to be 62900 $L^{-1}\cdot mol^{-1}\cdot cm^{-1}$ in water. This was used with the Lambert-Beer equation to determine the dye molarity in a 3.1 µM solution of **PIBMA$_{[389]}$-CD$_{[85]}$-Cy5$_{[x]}$ (7)**. Dividing the dye molarity by 3.1 µM thus provided an average number of 1.53 Cy5 molecules per polymer; for clarity of illustration and calculation this was rounded to 2 Cy5 dyes per polymer.

3.3 Quantification of CL307 (the adjuvant) on polymer

The molar extinction coefficient of CL307 was estimated as previously described;[2] briefly: a weighed quantity of CL307 was dissolved in water to yield a 1 mM stock solution. From this stock a dilution range from 7.5 µm to 0.25 µm was made in triplicate, of which the absorption was measured at Abs_{max} = 298 nm. Using linear regression, the molar extinction coefficient was determined to be 5200 $L^{-1}\cdot mol^{-1}\cdot cm^{-1}$ in water. This was used with the Lambert-Beer equation to determine the CL307 molarity in a 0.5 µM solution of **PIBMA$_{[389]}$-CD$_{[85]}$-Cy5$_{[2]}$-CL307$_{[x]}$ (9)**. Dividing the CL307 molarity by 0.5 µM thus gave an average number of 58 CL307 molecules per polymer.

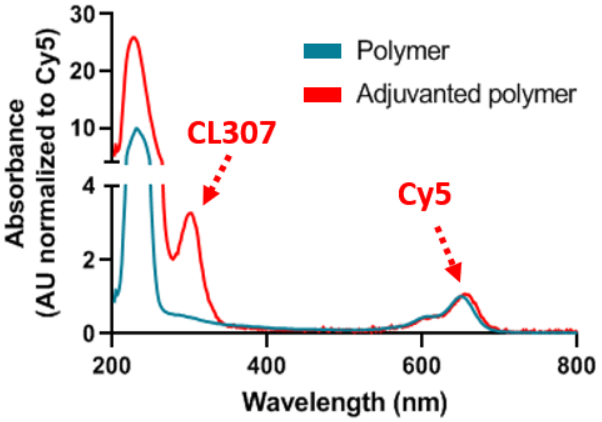

Figure S2: Confirmation of CL307 presence on the polymer was obtained using UV-Vis spectroscopy by measuring a solution of PIBMA$_{[389]}$-CD$_{[85]}$-Cy5$_{[2]}$-CL307$_{[58]}$-NEtOH$_{[633]}$ (9) in water (red line). This shows amide-bond absorption around 214 nm, absorption of CL307 around 300 nm and Cy5 absorption around 650 nm. As a control, PIBMA$_{[389]}$-CD$_{[85]}$-Cy5$_{[2]}$ (7) was also measured (turquoise line) showing only amide-bond and Cy5 absorption around 214 nm and 650 nm, respectively.

3.4 NMR

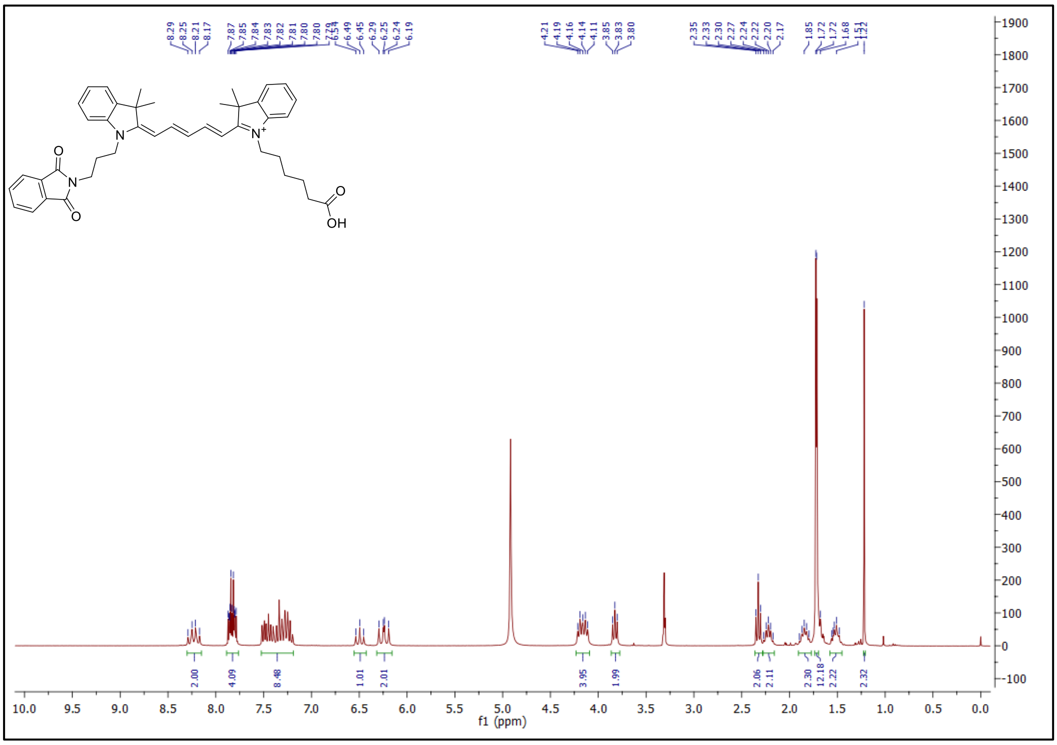

Figure S3: ^{1}H NMR (300 MHz, MeOD) of Phth-Cy5-COOH (4)

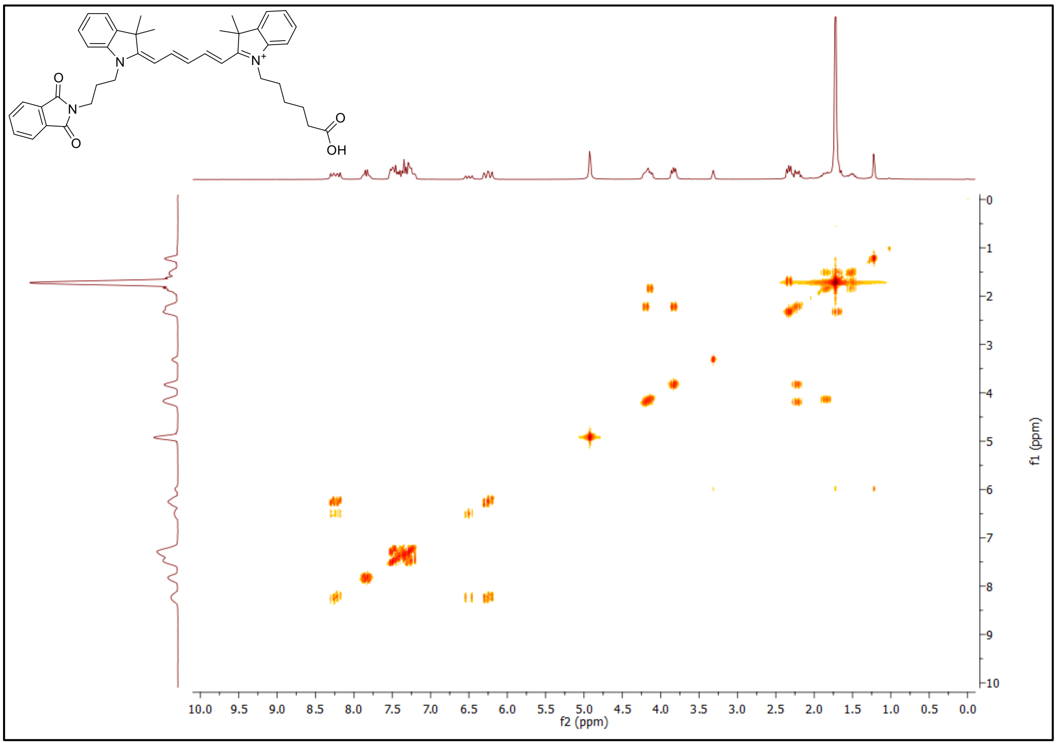

Figure S4: ¹H NMR COSY (300 MHz, MeOD) of Phth-Cy5-COOH (4)

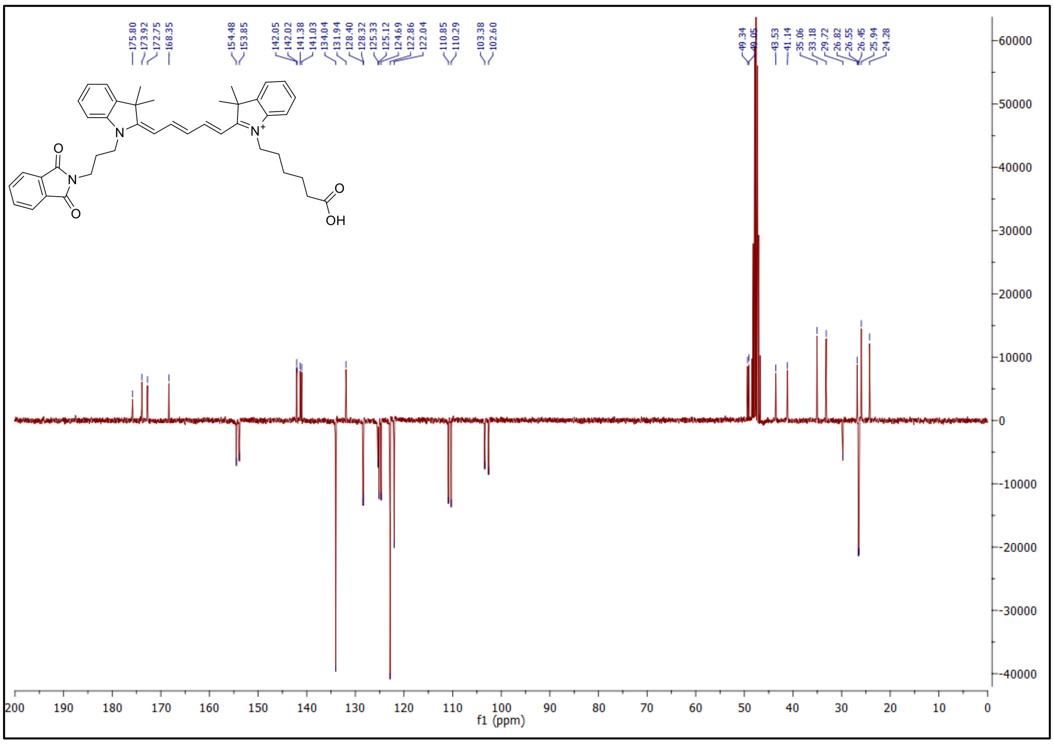

Figure S5: ¹³C APT NMR (75 MHz, MeOD) of Phth-Cy5-COOH (4)

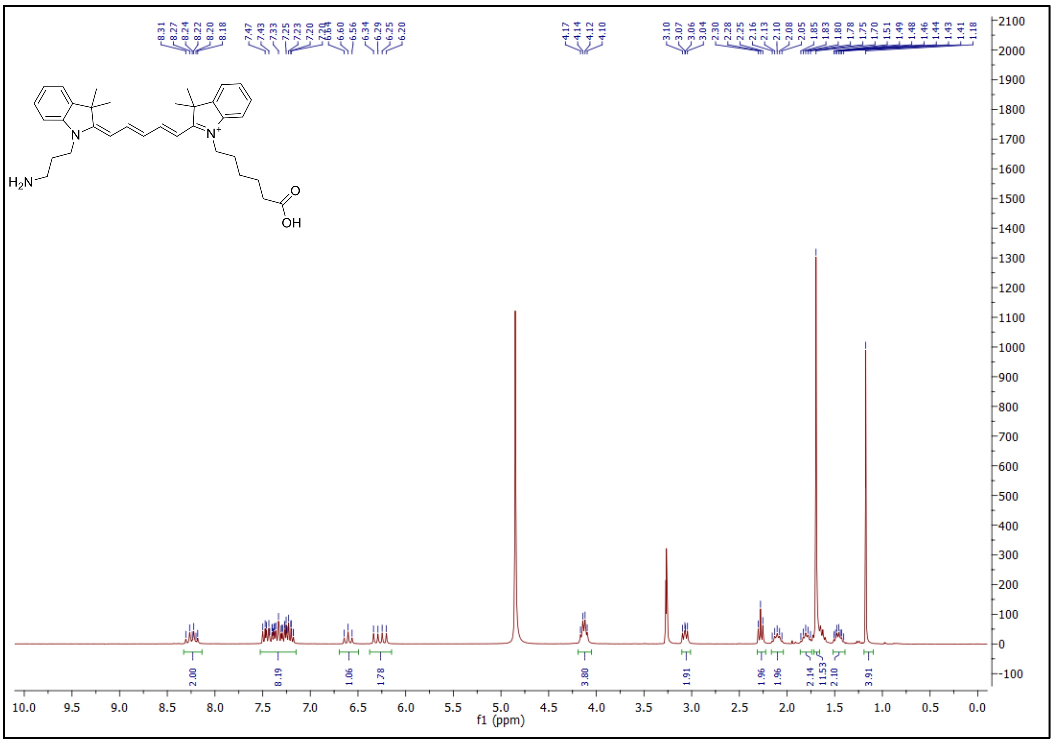

Figure S6: ^{1}H NMR (300 MHz, MeOD) of NH_2-Cy5-COOH (5)

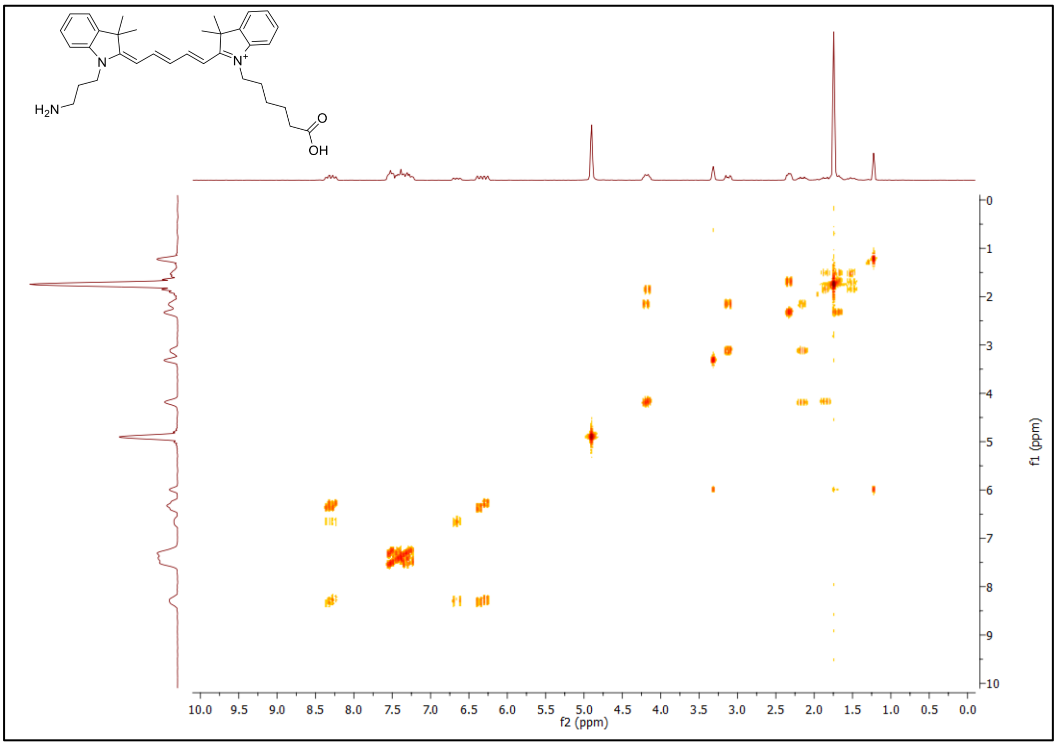

Figure S7: ^{1}H NMR COSY (300 MHz, MeOD) of NH_2-Cy5-COOH (5)

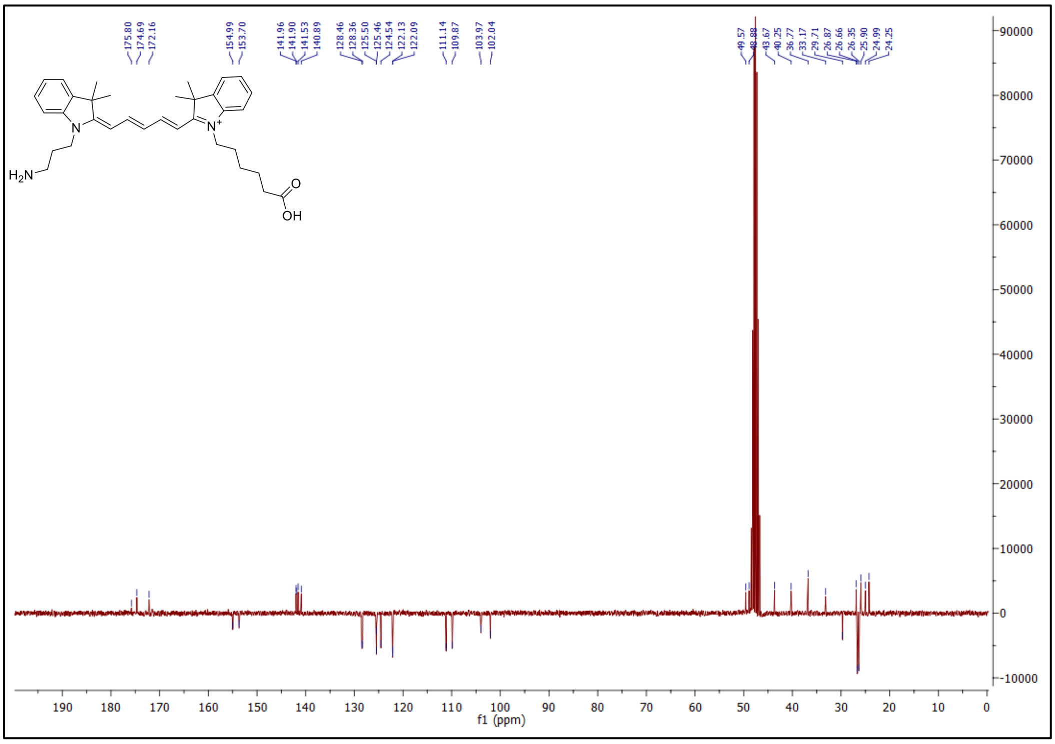

Figure S8: ^{13}C APT NMR (75 MHz, MeOD) of NH_2-Cy5-COOH (5)

3.5 HPLC

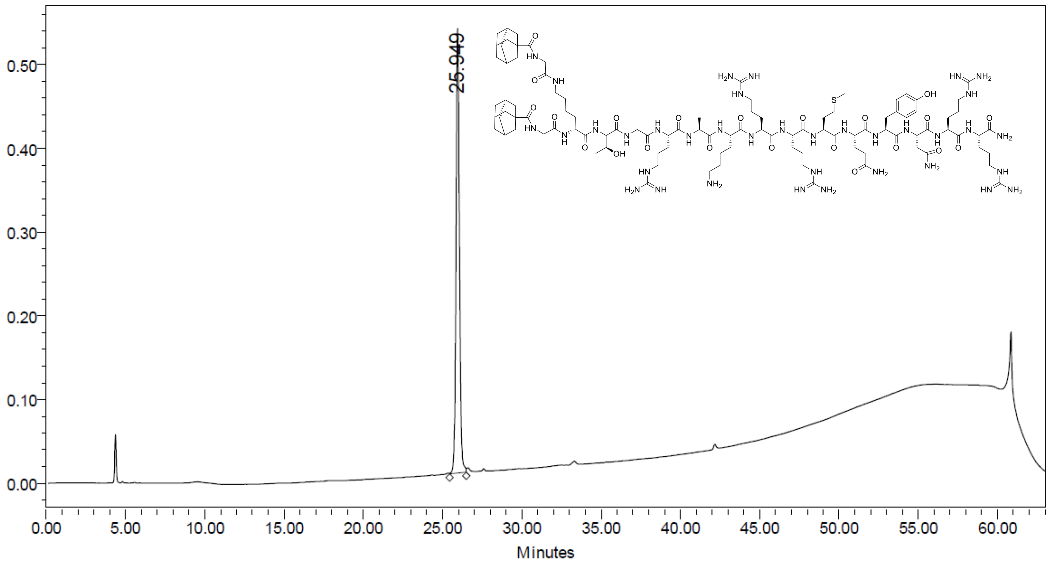

Figure S9: HPLC (220 nm) of *UBI-Lys(Gly-Ad)$_2$ (1)*

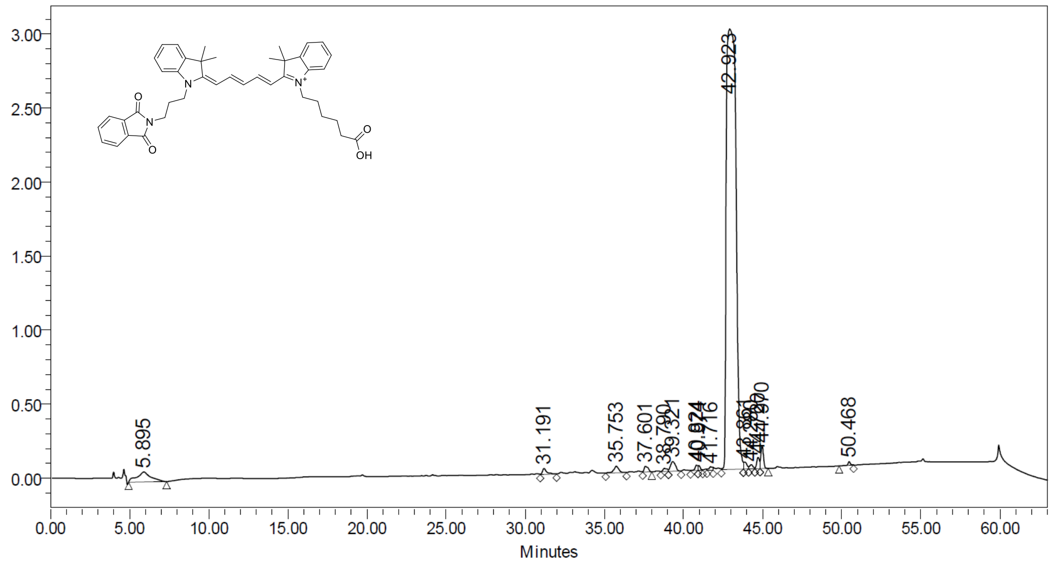

Figure S10: HPLC (220 nm) of Phth-Cy5-COOH (4)

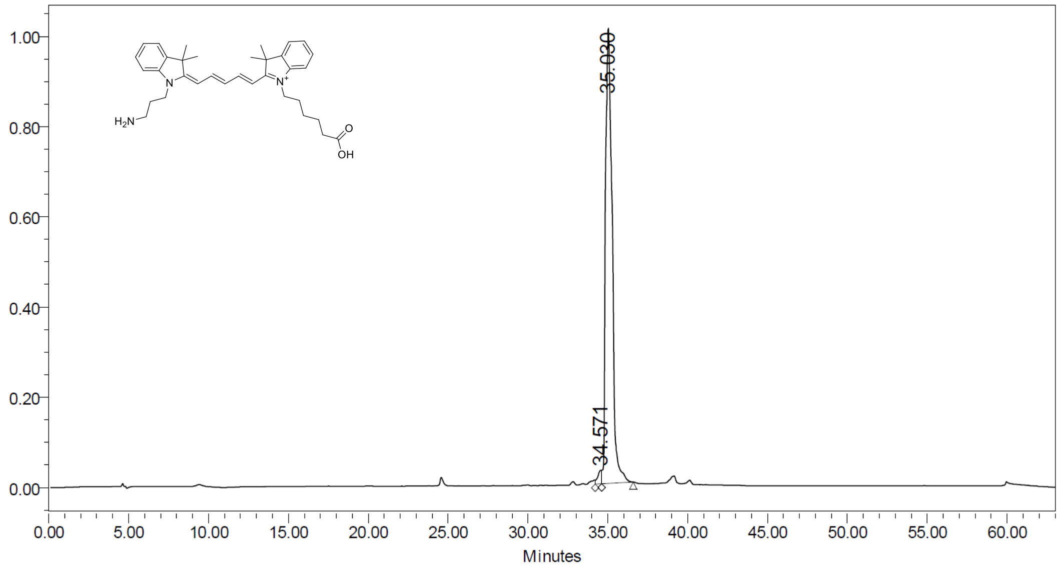

Figure S11: HPLC (220 nm) of NH_2-Cy5-COOH (5)

B. Additional figures

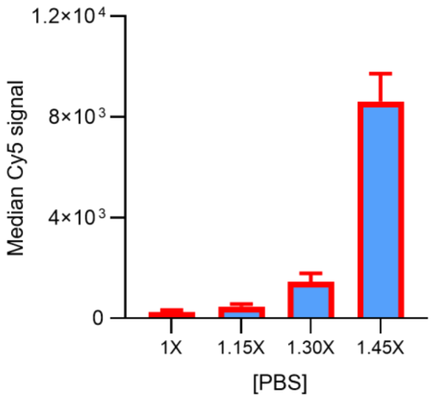

Figure S12: Complexation of adjuvant-bearing polymers onto *S. aureus* at varying PBS concentrations. Median Cy5 signal (y-axis) of complexed adjuvant-bearing polymer at differing concentrations of PBS (x-axis) during complexation.

References

[1] D. Oushiki, H. Kojima, T. Terai, M. Arita, K. Hanaoka, Y. Urano, T. Nagano, *J Am Chem Soc* **2010**, *132*, 2795-2801.
[2] A. W. Hensbergen, T. Buckle, D. M. van Willigen, M. Schottelius, M. M. Welling, F. A. van der Wijk, T. Maurer, H. G. van der Poel, G. van der Pluijm, W. M. van Weerden, H. J. Wester, F. W. B. van Leeuwen, *J Nucl Med* **2020**, *61*, 234-241.
[3] M. T. Rood, S. J. Spa, M. M. Welling, J. B. Ten Hove, D. M. van Willigen, T. Buckle, A. H. Velders, F. W. van Leeuwen, *Sci Rep* **2017**, *7*, 39908.
[4] N. Fattahi, M. Ayubi, A. Ramazani, *Tetrahedron* **2018**, *74*, 4351-4356.
[5] N. Duszenko, D. M. van Willigen, M. M. Welling, C. M. de Korne, R. van Schuijlenburg, B. M. F. Winkel, F. W. B. van Leeuwen, M. Roestenberg, *ACS Infect Dis* **2020**, *6*, 1734-1744.
